# Repeat Composition of CenH3-chromatin and H3K9me2-marked heterochromatin in Sugar Beet (*Beta vulgaris*)

**DOI:** 10.1186/s12870-016-0805-5

**Published:** 2016-05-26

**Authors:** Teresa Kowar, Falk Zakrzewski, Jiří Macas, Andrea Kobližková, Prisca Viehoever, Bernd Weisshaar, Thomas Schmidt

**Affiliations:** Department of Plant Cell and Molecular Biology, TU Dresden, Dresden, D-01062 Germany; Biology Centre ASCR, Institute of Plant Molecular Biology, Branišovská 31, Česke Budějovice, CZ-37005 Czech Republic; CeBiTec & Faculty of Biology, Bielefeld University, Universitätsstr. 25, Bielefeld, D-33615 Germany

**Keywords:** Centromere, CenH3, H3K9me2, Heterochromatin, Repeats, *Beta vulgaris*, ChIP-Seq

## Abstract

**Background:**

Sugar beet (*Beta vulgaris*) is an important crop of temperate climate zones, which provides nearly 30 % of the world’s annual sugar needs. From the total genome size of 758 Mb, only 567 Mb were incorporated in the recently published genome sequence, due to the fact that regions with high repetitive DNA contents (e.g. satellite DNAs) are only partially included. Therefore, to fill these gaps and to gain information about the repeat composition of centromeres and heterochromatic regions, we performed chromatin immunoprecipitation followed by sequencing (ChIP-Seq) using antibodies against the centromere-specific histone H3 variant of sugar beet (CenH3) and the heterochromatic mark of dimethylated lysine 9 of histone H3 (H3K9me2).

**Results:**

ChIP-Seq analysis revealed that active centromeres containing CenH3 consist of the satellite pBV and the Ty3-*gypsy* retrotransposon Beetle7, while heterochromatin marked by H3K9me2 exhibits heterogeneity in repeat composition. H3K9me2 was mainly associated with the satellite family pEV, the Ty1-*copia* retrotransposon family Cotzilla and the DNA transposon superfamily of the En/Spm type. In members of the section *Beta* within the genus *Beta*, immunostaining using the CenH3 antibody was successful, indicating that orthologous CenH3 proteins are present in closely related species within this section.

**Conclusions:**

The identification of repetitive genome portions by ChIP-Seq experiments complemented the sugar beet reference sequence by providing insights into the repeat composition of poorly characterized CenH3-chromatin and H3K9me2-heterochromatin. Therefore, our work provides the basis for future research and application concerning the sugar beet centromere and repeat-rich heterochromatic regions characterized by the presence of H3K9me2.

**Electronic supplementary material:**

The online version of this article (doi:10.1186/s12870-016-0805-5) contains supplementary material, which is available to authorized users.

## Background

Providing approx. 30 % of the world’s annual sugar demands, *Beta vulgaris subsp. vulgaris* (hereinafter referred to as sugar beet) is an important crop of the temperate climate zones. It possesses 2n = 18 chromosomes, an estimated genome size of 758 megabases (Mb) [1] and a repeat content of 63 % [2]. The annual or biennial plant belongs to the genus *Beta*, within the order *Caryophyllales* and the family *Amaranthaceae*. Recently, the genome sequence has been published and accounts for 567 Mb [3]. The genus *Beta* comprises the three sections *Beta*, *Corollinae* and *Nanae*. Species of the section *Beta* are widely distributed along the Mediterranean, central and northern Atlantic coastlines [4]. In contrast, *Nanae* is endemic to Greece and contains only one species (*Beta nana*). Species of the sections *Corollinae* are found in the Mediterranean area as well as in south-west Asia. The closely related wild beet genus *Patellifolia* (formerly known as section *Procumbentes* within the genus *Beta*) comprises the species *Patellifolia webbiana*, *Patellifolia procumbens* and *Patellifolia patellaris* [5, 6]. While *P. webbiana* and *P. procumbens* possess a diploid genome with 2n = 18 chromosomes, *P. patellaris* is tetraploid [7, 8].

Long terminal repeat (LTR) retrotransposons were the most prominent class of repetitive elements detected in the genome assembly of sugar beet. In particular, Ty3-*gypsy* retrotransposons were predominantly found in centromeric and pericentromeric regions [3]. The most abundant satellites pBV and pEV are only fragmentarily included in the assembly. Both satellites are organized in large arrays and serve as cytogenetic markers for the heterochromatic regions of chromosomes of the genus *Beta* [9–11]. Technical barriers of second generation sequencing (reviewed in [12]) hamper the complete arrangement of repetitive sequence elements (particularly satellite DNAs) in the genome assembly. Subsequently, this incompleteness contributes to the deviation of the estimated genome size from the size of the assembled reference sequence. As a result, heterochromatic regions possessing a high proportion of repetitive elements lack characterization and assignment, which also limits subsequent analysis of epigenetic modifications [13]. Heterochromatin is influenced by a variety of epigenetic modifications, including DNA methylation, the modification of single histones or the incorporation of specific histone variants (reviewed in [14]). A histone variant of outstanding interest is the centromere specific histone H3 variant, described as CenH3 in plants, CID in Drosophila, and CenP-A in mammals [15]. CenH3 marks centromeric chromatin and is indispensable for proper centromere function. Being responsible for kinetochore formation [16–19]⁠ CenH3 nucleosomes enable the centromere to act as early guide in cell division during mitosis and meiosis. Thus, CenH3 serves as a hallmark for active centromeres in many plant and animal species [20–25]. While the CenH3 function remains similar in all species studied so far, the protein including its DNA binding domain differs even between closely related species [15, 26, 27]. CenH3 proteins differ from canonical H3 in the N-terminal end and the loop1 region, which is part of the C-terminal histone fold domain [26, 28]. In consequence, CenH3 antibodies are raised against N-terminal ends of CenH3 proteins to produce species-specific CenH3 antibodies. The C-terminus, including the histone fold domain, is more conserved. For plants it is known that CenH3 is preferentially associated with satellite DNA often intermingled with Ty3-*gypsy* retrotransposon elements [28–30].

Next to CenH3-chromatin, pericentromeric-, intercalary- and subtelomeric regions exhibit heterochromatic features [31]. In plants, heterochromatin is mainly characterized by the dimethylation of lysine 9 of histone H3 (H3K9me2) [13, 32–34]. H3K9me2 is incorporated in genomic regions rich in DNA methylation – another hallmark of heterochromatin [34, 35]. Furthermore, H3K9me2 was detected in genic regions or at transposons [34]. In addition, multiple studies demonstrate that H3K9me2-heterochromatin may also be present at CenH3 occupied chromatin [32, 36, 37]. Therefore, H3K9me2 represents a hallmark of heterochromatin, on the scale of large genomic regions down to smaller sites in genic regions as well as in active centromeres. Sugar beet heterochromatin can be distinguished in large intercalary blocks on both chromosome arms, in pericentromeric and centromeric heterochromatin as well as in smaller subtelomeric and interspersed heterochromatic sites, which are visible as strong DAPI staining in metaphase spreads (e.g. Fig. 2 in Dohm et al. 2014 [3]). Fluorescence-*in situ*-hybridization (FISH) experiments demonstrated that the satellite pEV and the Ty1-*copia* retrotransposon family Cotzilla are largely amplified in intercalary heterochromatin [13, 38] while pericentromeric and centromeric heterochromatin largely consist of the pBV satellite and the Ty3-*gypsy* retrotransposon family Beetle [13, 39]. The subtelomeric heterochromatic regions are characterized by the presence of the satellite family pAV [40]. Small dispersed heterochromatic spots consist of a variety of different repetitive elements including satellite DNAs, DNA transposons and LTR and Non-LTR retrotransposons [41–44]. In interphase nuclei it has been shown that sugar beet heterochromatin is characterized by the presence of H3K9me2. Very strong signals were detected in intercalary heterochromatin. Pericentromeric and centromeric heterochromatin as well as interspersed heterochromatic sites are characterized by very faint signals of H3K9me2 only [13]. Furthermore, intercalary, pericentromeric and centromeric heterochromatin is characterized by lower levels of DNA methylation compared to adjacent genomic regions. This might be due to the presence of the large AT-rich satellite arrays in which most of the cytosine occur in the asymmetric CHH (H = A, C, T) motif with only low chances to be methylated [13]. The same can be observed for smaller dispersed satellite arrays [42]. Interestingly, satellites are transcribed and transcripts may be processed into small RNAs mostly 24 nucleotides in size which might point to a functional role in heterochromatin maintenance [13, 42].

Chromatin immunoprecipitation followed by high-throughput sequencing (ChIP-Seq) is the method of choice for investigating sequence composition of genomic regions associated with specific types of chromatin (reviewed in [45]). Very important work has already been conducted in maize to analyze the DNA sequence composition of CenH3 chromatin and to investigate the evolution of centromeres and centromeric repeats [46–53]. However, the application of ChIP-Seq for the characterization of repeat-rich regions like centromeres is often hampered by the lack of a proper reference, because these regions are only poorly represented in genome assemblies. An alternative approach introduced by Neumann *et al.* [54] and Gong *et al*. [55] employs the output of graph-based repeat clustering analysis as the reference. In this method, repeat composition of the genome is first analyzed by clustering of unassembled shotgun genomic reads into groups of frequently overlapping sequences (clusters) representing individual families of repetitive elements [56]. The clustering is executed by RepeatExplorer, a computational software pipeline that also performs various supplementary analyzes supporting the repeat annotation [57]. The annotated output of the clustering analysis can then be used as a reference for similarity-based mapping of ChIP-Seq reads. Repeats enriched in the selected type of chromatin are identified by elevated proportions of reads from ChIP versus control. We employed this approach on CenH3- and H3K9me2 ChIP-Seq data to elucidate the complete sequence composition of active centromeres marked by CenH3- and H3K9me2-heterochromatic regions of the sugar beet genome.

## Results

### Clustering of genomic reads gives insights into the total repeat content of the sugar beet genome and provides a reference for ChIP-Seq read mapping

**Fig. 1:**
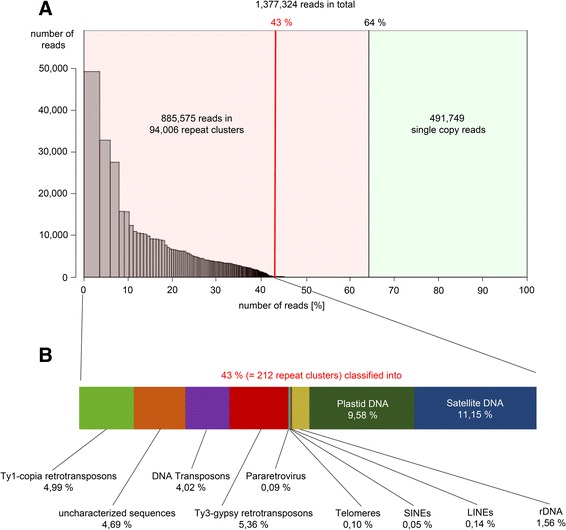
Genomic clusters of sugar beet. **a** Summary of the clustering analysis. Illumina reads were integrated into genomic clustering resulting in 94,006 repeat clusters. Reads in clusters situated left from the red line were analyzed in detail (=212 clusters making up 43 % of the genome). **b** Proportions of different repeats in the 212 genomic reference clusters

To provide a reference repeat data base to analyze the repeat composition of the ChIP-Seq data, paired-end Illumina raw reads originating from the sugar beet sequencing project [3] were used for repeat clustering. Reads were processed and clustered using the RepeatExplorer software [57]. Clustering of 1.4 million reads resulted in 94,006 clusters and 491,749 single/non-clustered reads (Fig. 1a). The 94,006 clusters represent all repetitive families and subfamilies of the sugar beet genome which account in total for 64 % of the analyzed reads (Fig. 1a). For a detailed characterization of the most abundant repeat families, the 212 most highly-repetitive clusters were annotated. Each of these clusters represents at least 0.01 *%* of the sugar beet genome. Subsequent annotation of ChIP-Seq reads is based on these first 212 clusters (accounting in total for 43 % of genomic reads), which are further referred to as the genomic reference clusters of sugar beet. For complete cluster information see Additional file 1: Table A1. Within the 212 clusters satellites are the most abundant repeat group, followed by plastid DNA (11.15 % and 9.58 %, respectively, Fig. 1b). Retrotransposons of the Ty3-*gypsy* type are highly abundant occupying 5.36 % of the genome and Ty1-*copia* retrotransposons make up 4.99 % of the genome. Ribosomal genes (rDNA) represent 1.56 % of the genome while Pararetroviruses, telomeric DNA, long interspersed nuclear elements (LINEs) and short interspersed nuclear elements (SINEs) show genome proportions of ≤1 %.

The presented clustering approach enables a description of the repetitive genome content, since it does not depend on any genome assembly. Instead, all related sequences found in the reads are grouped to *in silico* repeat clusters. The data obtained by the clustering approach is useful to gain information about sequence composition and genome proportion of repeats which are only fragmentary included in the reference sequence. Therefore, the combination of the repeat annotation of the reference sequence together with the clustering repeat data draws a more realistic picture of the repeat landscape within the sugar beet genome. In Table 1, the clustering approach and results from the genome assembly (RefBeet 1.1 [3]) are compared. Remarkably, highly abundant repeats such as tandem repeats, e.g. the satellites pBV and pEV, are strongly underrepresented in the genome assembly covering only 0.27 % and 0.17 %, respectively. However, the clustering of genomic reads resulted in a genome proportion of 6.17 % and 3.58 % for pBV and pEV. This is a more realistic description of the proportion of these two repeats in the sugar beet genome and is consistent with a renaturation kinetics study which showed that both satellites are the most abundant repeat families in the sugar beet genome. pBV and pEV make up 32.8 % and 29.3 %, respectively, of the highly repetitive *c*_*0*_*t-1* DNA fraction demonstrating that both satellites are largely amplified in the genome [10]. The same is observed for selected retrotransposon families, members of the Ty3-*gypsy* retrotransposons Beetle and Bongo3 (e.g. Beetle7 covers 0.45 Mb of the RefBeet 1.1 and 4.89 Mb in the clustering approach) and members of Ty1-*copia* retrotransposons. The Cotzilla family [38] is the most prominent example of Ty1-*copia* elements. Cotzilla elements cover 22.77 Mb (3.02 %) of the genome in clustering data, but was described in the genome assembly to make up only 9.75 Mb (1.72 %). Table 1 exemplarily shows repeats demonstrated to constitute the sugar beet heterochromatin. It describes the higher sensitivity of the clustering approach towards repeat proportions and quotes an example to replenish poorly described genome regions within the assembly. For certain types of repeats, RepeatExplorer can produce smaller size estimates compared to the genome assembly annotation. This can most likely happen to repeats that are relatively less abundant and highly variable in their sequences, and therefore will produce multiple small clusters in the RepeatExplorer output which may be below the threshold for annotation.

**Table 1:** Genomic repeat contents in genome assembly and after clustering in comparison

		Genome Assembly		RepeatExplorer (758 Mb)			ChIP enrichment	
		RefBeet 1.1 (567 Mb)						
		genome proportion	size	genome proportion	size	Δ		
		[%]	[Mb]	[%]	[Mb]	[Mb]	CenH3	H3K9me2
Repeat								
Superfamily								
	family							
Ty3-*gypsy* retrotransposons		7.61	43.15	4.37	33.09	−10.23	0.8–7.81	0.42–3.38
	Beetle2	0.12	0.68	0.48	3.67	2.99	3.00	2.01
	Beetle4	0.00	0.00	0.10	0.74	0.74	4.54	2.07
	Beetle7	0.08	0.45	0.65	4.92	4.47	5.34	1.58
	Total Beetle	0.50	2.84	1.25	9.50	6.66	3.0–5.34	0.75–2.07
	Bongo3	2.02	11.45	1.90	14.42	2.96	0.28	0.63–1.62
Ty1- *copia* retrotransposons		4.56	25.86	4.99	37.82	11.97	0.11–0.49	0.8–2.37
	Cotzilla	1.72	9.75	3.02	22.89	13.14	0.25–0.15	0.8–1.83
	Salire	0.34	1.93	0.49	3.71	1.79	0.16	1.83
	Patty	0.18	1.02	0.10	0.72	−0.30	0.11	2.37
*ENV*-like retrotransposons		4.05	22.96	2.14	16.22	−6.74	0.10–0.42	1.01–2.55
	Elbe family	3.01	17.07	2.03	15.41	−1.66	0.10–0.42	1.01–2.43
LINE		3.77	21.38	0.14	1.06	−20.31	0.16	2.65
Tandem Repeat		3.31	18.77	11.15	84.52	65.75	0.08–3.22	0.33–3.61
	pBV	0.27	1.53	6.17	46.75	45.21	2.34–3.22	0.87–1.2
	pEV	0.17	0.96	3.58	27.14	26.17	0.13	2.6
	Niobe	0.02	0.11	0.06	0.46	0.35	0.14	1.81
	BvSat04	0.03	0.17	0.03	0.19	0.02	0.08	3.61
DNA transposons		2.07	11.74	3.93	29.79	18.05	0.11–1.02	0.25–3.15
	Mutator	0.00	0.00	2.01	15.24	15.24	0.11–1.02	0.26–3.15
	En/Spm (CACTA superfamily)	0.00	0.00	0.89	6.78	6.78	0.12–0.17	1.84–2.14
Helitron		0.56	3.18	0.10	0.76	−2.42	0.12	1.51
SINE		0.49	2.78	0.05	0.38	−2.40	0.31–0-64	0.37–0.46
rDNA		0.15	0.85	1.56	11.82	10.97	0.17–0.42	0.47–1.05
Pararetrovirus		0.06	0.34	0.09	0.68	0.34	0.5	1.55
Unclassified Dispersed Repeats		0.08	0.45	0.00	0.00	−0.45	-	-
plastid DNA		0.00	0.00	9.58	72.62	72.62	1.77–3.09	1.62–1.79
Unknown		6.28	35.61	4.69	35.55	−0.06	0.08–4.10	0.23–3.64

### ChIP-Seq data reveal repeat composition of sugar beet CenH3- and H3K9me2-marked chromatin

To study the sugar beet centromeric chromatin and H3K9me2-heterochromatin thoroughly on the level of the underlying DNA sequences, we carried out chromatin immunoprecipitation followed by DNA sequencing (ChIP-Seq) using antibodies against CenH3 and H3K9me2. Ten million randomly selected ChIP-Seq and control input reads were mapped to genomic reference clusters using BLAST. Each read was assigned to maximally one cluster, and the ratio of ChIP to input read numbers was calculated for all reference clusters. The ChIP/Input ratio > =1.5 was chosen as a threshold for considering the corresponding repeat enriched in the ChIP sample. This was well above the values obtained for repeats which due to their known positions on chromosomes could be used as controls. For example, the satellite pEV located in intercalary heterochromatin [13] and 45S rDNA repeats which are also absent from the centromeres showed no enrichment in CenH3 ChIP experiments (ChIP/Input ratios of 0.13 and 0.34, respectively). When using H3K9me2 antibody, the ChIP/Input ratio for 45S rDNA sequences was 0.88. On the other hand, ChIP-Seq data confirmed expected association of the chromovirus Beetle7 [39] and the satellite pBV [13, 58] with CenH3 chromatin and Cotzilla and pEV repeats [38] with heterochromatin marked by H3K9me2 (Table 1). For the chromovirus Beetle7 [39] and the satellite pBV [13, 58] small enrichment factors of 1.58 and 1.1–1.2 were detected, respectively, indicating to a presence of H3K9me2 in centromeres. Overall, 46 and 96 repeat clusters were found enriched in CenH3 and H3K9me2 ChIP reads, respectively. Cluster annotation was performed to the level of repeat clades and, if possible, to family level. The enrichment factor accounts for the specificity of a repetitive element to the specific chromatin region. In turn, the information about composition and abundance of repetitive sequences in the genomic region is found in the proportion of read counts to individual repeat classes, clades, and families.

**Fig. 2:**
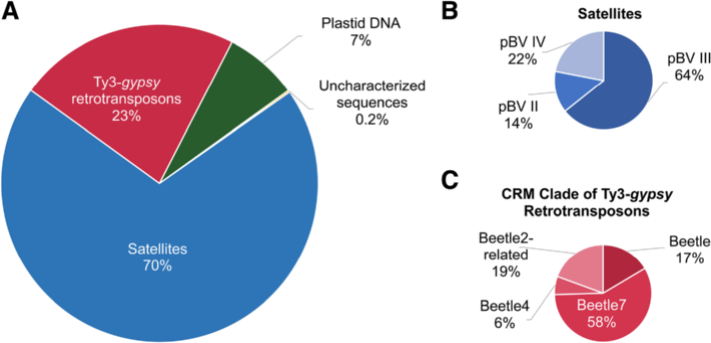
Sequence composition of CenH3-chromatin (CenH3 ChIP-Seq). Proportional read counts assigned to repeat groups (**a**), and more specifically to clades and families (**b** and **c**). The repeat proportions reflect repeat abundance within sugar beet centromeric sequences. Read counts were normalized to the overall read amount introduced into the clustering approach. **a** The active centromeres of sugar beet consist mainly of pBV satellite DNA, Ty3-*gypsy* retrotransposons and of plastid DNA. Only 0.2 *%* are uncharacterized repeats, consisting of either putative unknown centromeric sequences or truncated repeats. **b** Satellites of only the pBV family constitute the active centromeres. The most abundant subfamily is pBV_III. **c** Ty3-*gypsy* retrotransposons in the active centromeres are exclusively consisting of Beetle families with Beetle7 being the most prominent Ty3-*gypsy* retrotransposon. Note, that sequences assigned to Beetle2 are most likely sugar beet specific Beetle2 homologs

The enriched clusters in CenH3 ChIP-Seq experiments were divided into four repeat groups (Fig. 2a and Additional file 2: Table A2), namely satellites (70 *%* of reads hit to satellite clusters), Ty3-*gypsy* retrotransposons (23 *%*), plastid DNA (7 *%*) and uncharacterized sequences (0.2 %). In satellites, which exhibit the most abundant repeat type in centromeric chromatin, only copies of three out of six pBV subfamilies were found (Fig. 2b). Ty3-*gypsy* retrotransposons consist exclusively of the Beetle family (Fig. 2c) with Beetle7 accounting for 58 *%*. To exclude the putative existence of wild beet-specific Beetle2 sequences [59, 60], a similarity search in the sugar beet reference sequence (RefBeet 1.1) using the 774 bp LTR sequence of Beetle2 was conducted confirming the absence of Beetle2 sequences in sugar beet. However, because of their assignment to Beetle2 clusters these sequences were designated Beetle2-related. A very small proportion (0.2 *%*) of the reads has not been annotated yet and most likely represent either truncated repeating units or unknown centromere-specific repeats in sugar beet (Fig. 2a).

**Fig. 3:**
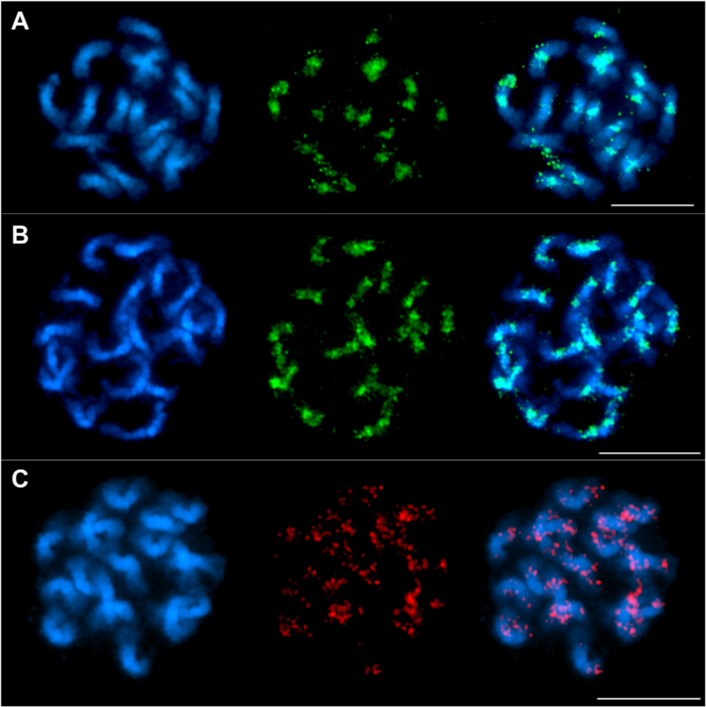
FISH using ChIP-DNA and plastid DNA probes on sugar beet metaphase chromosomes Chromosomes were counterstained with DAPI (blue signals) revealing chromosome morphology. **a** and **b** Location of immunoprecipitated DNA is shown. Probes were labelled with digoxigenine and detected with antidigoxigenine coupled to FITC (green signals). **a** CenH3 ChIP-DNA probe detects centromeric regions. Bar = 5 μm. **b** H3K9me2 ChIP-DNA signals are distributed along the chromosome with stronger signals in the major intercalary heterochromatic region of each arm. Note, that signal strength is reduced in centromeric regions. Signals in centromeric regions are due to sequences occurring in association with both, CenH3 in the centromere and H3K9me2, e.g. sequences of Beetle4. The signals may be also due to the presence of H3K9me2 in active centromeres. Distal euchromatin shows depletion of signals. Bar = 5 μm. **c** Localization of plastid DNA on sugar beet chromosomes using a biotin dUTP-labelled plastid DNA probe detected by streptavidine-Cy3 (red signals). Plastid DNA sequences are found dispersed on all 18 chromosomes without restriction to particular chromosome regions. Bar = 5 μm

To validate the specificity of DNA sequences identified by immunoprecipitation towards sugar beet CenH3-chromatin, FISH experiments were conducted using probes generated of CenH3 ChIP-DNA (Fig. 3a). CenH3 ChIP-DNA largely hybridized in heterochromatic centromeric and pericentromeric regions of all chromosomes with variable but strong intensity.

**Fig. 4:**
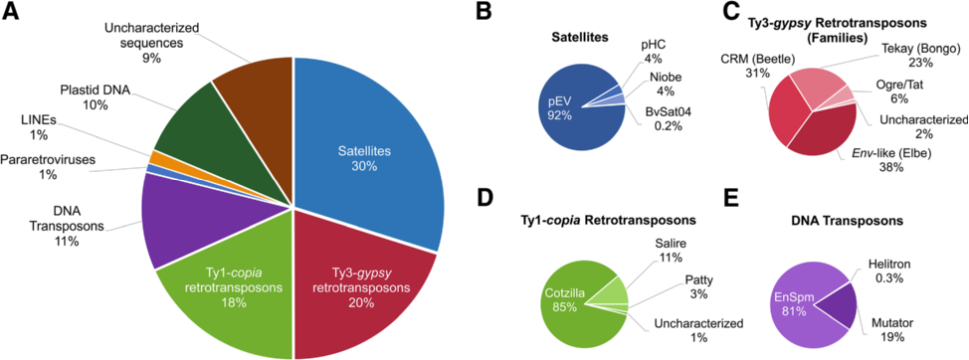
Sequence composition of H3K9me2-heterochromatin (H3K9me2 ChIP-Seq). Proportional read counts assigned to individual sequence clusters are divided into main repeat groups (**a**), and subsequently into clades and families (**b** and **c**). The repeat proportions reflect their abundances within sugar beet H3K9me2-heterochromatin. Read counts are normalized to the overall read amount introduced into the clustering approach. **a** H3K9me2-heterochromatin displays heterogeneity in repeat composition. Satellite DNA is the most abundant repeat and is followed by LTR retrotransposons (Ty3-*gypsy* as well as Ty1-*copia* retrotransposons). **b** The family pEV is the prevalent satellite found in H3K9me2-heterochromatin. Regarding the abundances, the minisatellite BvSat04 and the satellites pHC, and Niobe contribute only partially to H3K9me2-heterochromatin sequences. **c** At least four clades of Ty3-*gypsy* retrotransposons are associated with H3K9me2-heterochromatin. 2 *%* are uncharacterized repeats and represent either putative unknown Ty3-*gypsy* sequences or truncated and/or recombined repeats in H3K9me2-heterochromatin. **d** The Cotzilla family predominantly contributes to the composition of H3K9me2-heterochromatin in Ty1-*copia* retrotransposons, while 1 *%* of the Ty1-*copia* sequences have not been characterized to date. **e** En/Spm is the prevailing DNA transposon superfamily followed by Mutator elements

The clusters enriched in H3K9me2 ChIP-Seq data were divided into eight repeat groups. In Fig. 4a the putative composition of H3K9me2-heterochromatin is shown. Similarly to CenH3, satellites are the most abundant repeats in H3K9me2-heterochromatin, comprising 30 *%*. At family level, the satellite pEV shows remarkably high proportions representing 89 *%* of all satellites (Fig. 4b). Both, Ty1-*copia* (18 *%*) and Ty3-*gypsy* (20 *%*) retrotransposons were highly enriched in H3K9me2-heterochromatin. Compared to the results in CenH3-chromatin, Ty3-*gypsy* retrotransposons are more diverse in H3K9me2-heterochromatin. Copies of five repeats are included, of which the *ENV*-like clade is the most abundant (Fig. 4c) and contains the sugar beet specific group Elbe [43]. The CRM clade is represented in 31 *%* of all detected Ty3-*gypsy* elements. Similar to CenH3, only members of the families Beetle2, Beetle4 and Beetle7 are found on CRM clade level (Additional file 3: Table A3). Among the Ty1-*copia* retrotransposons, three families were enriched (Fig. 4d), namely Cotzilla, Salire, and Patty. Repeat clusters related to the Cotzilla family of the SIRE clade represent 85 *%* of all Ty1-*copia* retrotransposons. These elements belong to the most abundant retrotransposon families in sugar beet (comprising up to 3 % of the genome) and were located preferentially in intercalary and pericentromeric heterochromatin [38]. DNA transposons are represented with 11 *%* in H3K9me2-heterochromatin, with the superfamilies En/Spm, Mutator and Helitron identified (Fig. 4e): The vast majority of transposons belongs to the transposon superfamily En/Spm. About 9 *%* of the reads were assigned to clusters that were not characterized so far, but still show enrichment in H3K9me2 ChIP-Seq data and might be unknown repeats specific for H3K9me2-heterochromatin (Fig. 4a). In ChIP-DNA-FISH experiments, H3K9me2 ChIP-DNA was detected by strong signals in intercalary heterochromatic regions with minor signals in most pericentromeric and centromeric regions as well as minor dispersed signals on all chromosomes (Fig. 3b). Hybridization to the outermost distal euchromatin (weak DAPI staining) of all chromosomes was not detected.

In summary, the enriched repeats were more diverse in H3K9me2 ChIP-Seq, both with respect to their quality and quantity. H3K9me2 ChIP-Seq detected 96 enriched clusters assigned to eight repeat types, while CenH3 ChIP-Seq detected 46 enriched clusters assigned to four repeat types.

To confirm integration of plastid DNA in the genome, hybridizations of plastid DNA probes to sugar beet mitotic metaphase chromosomes revealed dispersed signals along the 18 chromosomes (Fig. 3c). Notably, signals of plastid DNA were detected on both chromatides of most chromosomes arms with strong signals in subtelomeric and centromeric regions.

### Immunostaining reveals specificity of the sugar beet CenH3 antibody to species of the section *Beta* within the genus *Beta*

**Fig. 5:**
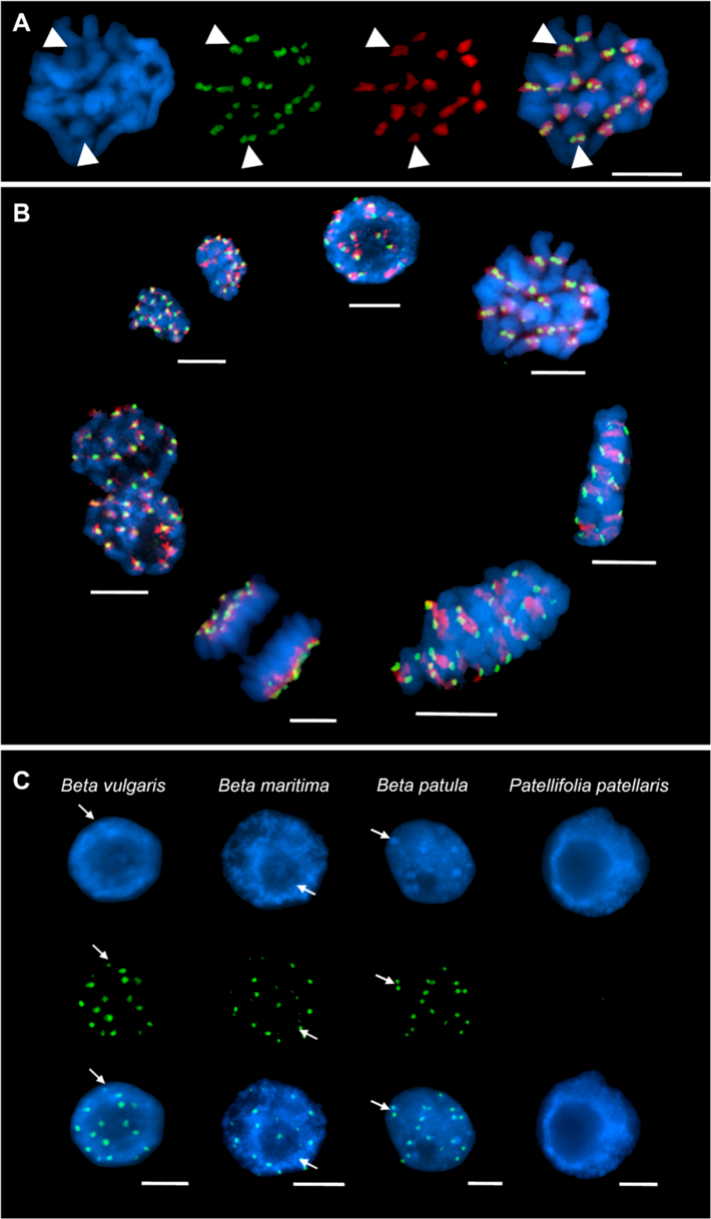
CenH3 immunofluorescence on *Beta* nuclei and FISH of pBV centromeric satellite on sugar beet. Nuclei were counterstained with 4’,6-diamine-2’-phenylindole-dihydrochloride (DAPI, blue signals) showing chromosome morphology. CenH3 binding was detected by the secondary antibody anti-rabbit coupled to fluorescein isothiocyanate (FITC; green signals). The pBV probe was labelled with biotin-11 UTP and detected by streptavidine-cyanine 3 (Cy3; red signals). Bar = 5 μm. **a** Localization of CenH3 on sugar beet metaphase chromosomes detected by immuno-FISH using the anti-bvCenH3 antibody and a centromeric satellite pBV probe as control. DAPI staining of an exemplarily chosen sugar beet metaphase shows chromosome morphology (left). Anti-bvCenH3 binding resulted in 36 signals, arranged in pairs corresponding to chromatides (middle left). 18 pBV signals (red) are detected and illustrate the centromeres (middle right). Overlay of all signals (right) reveals co-localization of two CenH3 signals within one large block of pBV on each chromosome (exemplarily indicated by arrowheads in all panels). **b** CenH3 localization during the sugar beet cell cycle. Anti-bvCenH3 binding displays CenH3 signals (green) in all mitotic cell cycle stages. 18 chromosomes are identified by anti-bvCenH3 binding on all chromosomes. Additional FISH using the centromeric satellite pBV probe (red) exhibits co-localization with CenH3 signals throughout the cell cycle. **c** CenH3 in species of the genera *Beta* and *Patellifolia*. Exemplarily chosen interphase nuclei of different *Beta* species are shown, including sugar beet*, B. maritima*, and *B. patula*, as well as P*. patellaris* belonging to *Patellifolia*. Areas exhibiting brighter DAPI staining indicate heterochromatic regions (exemplarily indicated by arrows, upper row). Distinct anti-bvCenH3 signals (green) were detected in sugar beet*, B. maritima* and *B. patula* in heterochromatic regions (arrows, middle and lower row). The 18 signals observed represent 2n = 18 chromosomes which is typical for diploid species of the section *Beta*. No signals were found on P*. patellaris* nuclei (genus *Patellifolia*)

Immunostaining using the antibody against sugar beet CenH3 (anti-bvCenH3) on metaphase nuclei resulted in 18 distinct and specific anti-bvCenH3 double-dot signals in sugar beet (Fig. 5a). Single CenH3 signals (green) and pBV regions (red) are clearly distinguishable on a representative metaphase (Fig. 5a). The CenH3 signals are arranged in pairs of two, corresponding to sister chromatids, while each pair is found in one pBV cluster. Most importantly, FISH experiments on interphase nuclei and mitotic chromosomes using the centromere-specific satellite probe pBV revealed a cell cycle stage-independent co-localization of bvCenH3 and pBV on sugar beet chromosomes (Fig. 5b).

The binding of anti-bvCenH3 was examined in sugar beet and additionally in related wild species of the genera *Beta* and *Patellifolia* to elucidate CenH3 conservation across *Beta* species- and genus borders (Fig. 5c). The species investigated included one member each of the same subspecies (*B. vulgaris subsp. maritima*, hereinafter referred to as *B. maritima*), of a different species (*B. patula*), and of the closely related genus *Patellifolia* (*P. patellaris*). Immunostaining using the anti-bvCenH3 on interphase nuclei resulted in 18 distinct and specific anti-bvCenH3 signals in sugar beet*, B. maritima* and *B. patula*, corresponding to the number of chromosomes in these species (2n = 18). Contrarily, for *P. patellaris* nuclei no bvCenH3 signals were observed. All anti-bvCenH3 signals are detected in intensively DAPI-stained centromeric heterochromatin, which implies that *Beta* centromeric regions form chromocenters in interphase nuclei (for sugar beet: Fig. 5c, arrows).

## Discussion

The recently published sugar beet reference sequence harbors 567 Mb [3], which accounts for approximately 75 % of the estimated genome size (758 Mb [1]). This difference is mainly due to technical barriers in the assembly of whole genome shotgun data predominantly generated with second generation sequencing technology [12]. The approach regularly fails to assemble highly repetitive genome portions, such as heterochromatic regions or centromeric chromatin harboring large arrays of tandemly repeated sequences and transposable elements. The clustering and BLAST approach of genomic and ChIP-Seq reads using the RepeatExplorer pipeline applied in this study resulted in a far more realistic description of the repetitive portion in the sugar beet genome. This procedure has been applied in other species, too, and has been proven to be efficient [54, 55]. Strikingly, most repeats identified as enriched in ChIP-Seq experiments (e. g. satellite DNAs) are clearly underrepresented in the sugar beet assembled genome sequence. As a result, our study complements the constitution of repetitive sequences comprising the CenH3-marked chromatin and H3K9me2-heterochromatin so far underrepresented in the reference sequence.

Immunostainings coupled to FISH experiments together with CenH3 ChIP-Seq data provide evidence on the direct interaction of the pBV satellite and CenH3. pBV was the only satellite family found to be enriched in the centromere. However, six variants of pBV are known [58, 61, 62]. The genomic abundance reported for the variant pBV_III (monomer size 327 bp) is reflected in CenH3 ChIP-Seq experiments, followed by pBV_II (monomer size 329 bp). The variant pBV_IV possesses the longest monomere size (385 bp) out of the pBV variants identified in ChIP-Seq data and is hypothesized to originate from pBV_III [63]⁠. The occurrence of only three out of six pBV variants in CenH3-chromatin, namely pBV_II, pBV_III, and pBV_IV, may be explained with high genome abundances of single variants and/or their individual monomer sizes potentially providing the basis for an optimal packaging of centromeric chromatin [64]⁠. Notably, the pBV variant VI (monomer size 573 bp), formerly designated as pRV [61]⁠, is not present in CenH3-chromatin. We conclude, that pBV_VI is not a repeat sequence in active centromeres although it is present in association with other pBV arrays [61]⁠. As it was detected throughout the mitotic cell cycle, the co-localization of CenH3 and pBV suggests an interaction between histone protein and satellite sequence [26, 29]⁠. In detail, recent studies demonstrated the active impact of long non-coding RNAs derived from repeated centromeric DNA on centromere function (reviewed in [65]). It is not the centromere sequence itself, but rather the sequence features which enables the maintenance of an epigenetic environment necessary for proper centromere function [65].

According to CenH3 ChIP-Seq data, additionally to pBV, Ty3-*gypsy* retrotransposons of the Beetle family constitute the sugar beet centromere, which is in accordance to the statements reported in Zakrzewski, Weber, and Schmidt (2013) based on FISH experiments [63]. The appearance of the satellite pBV and the Beetle retrotransposon families in sugar beet centromeric heterochromatin is typical for the compositions of plant centromeres [28, 29]. The combination of a satellite repeat in combination with retrotransposons as building blocks of centromeres was detected in many plants to date: e.g. in monocots (maize [66], barley [67], sugar cane [20] and rice [68, 69] as well as in dicots (Brassica species [70], *Arabidopsis thaliana* [71]*,* radish [72] and soybean [73].

According to ChIP-Seq data, two retrotransposon families of Beetle, Beetle4 and Beetle7, occur in sugar beet centromeres. These Ty3-*gypsy* retrotransposon families belong structurally to centromere-specific chromoviruses [56, 74, 75]. In total, seven Beetle families are known in the genera *Beta* and *Patellifolia* so far [39, 59, 63]. Their coding domains are highly conserved, while the flanking LTR regions are family-specific. Hence, the Beetle family covers species-specific members over various species. Beetle7 is a genuine centromeric retrotransposon family in sugar beet and was previously shown to be located within centromeric chromatin via FISH experiments [39]. Interestingly, a Beetle family related to Beetle2 was detected in addition to Beetle4 and Beetle7. Beetle2 is a wild beet-specific centromeric and pericentromeric repeat found in species of the genus *Patellifolia* (*P. procumbens*, *P. patellaris* and *P. webbiana* [59]). The existence of Beetle2 in sugar beet is most unlikely, since FISH as well as blot hybridization experiments using a Beetle2 LTR probe indicated specificity of Beetle2 sequences exclusively to wild beets [60]. The occurrence of clusters in sugar beet CenH3 ChIP-Seq data assigned to Beetle2 might be explained by the evolution of a species-specific, so far uncharacterized retrotransposon family after separation of sugar beet from wild species. Thus, a similar relation was recently suggested between Beetle1 from *P. procumbens* and its homolog in sugar beet, Beetle7*.* It is likely, that Beetle1 and Beetle7 share a common ancestor but acquired different LTRs during speciation [63]. Based on that knowledge, the annotation of the unknown retrotransposon as Beetle2 might be the result of high sequence similarity in the coding region between the wild-beet specific Beetle2 and its homolog in sugar beet. The characterization of the sugar beet Beetle diversity remains subject to future analyzes and might probably reveal an additional Beetle family specific for sugar beet centromeres. Another cluster annotated as centromere-specific chromovirus represents the highest enrichment of all repeats in CenH3 chromatin (Additional file 2: Table A2). Interestingly, this chromovirus is remarkably specific to the sugar beet centromere, but due to the lack of any LTR sequences could not be annotated to any family so far. 0.2 % of the enriched centromeric sequences are not annotated yet and remain without further information. The annotation of these sequences is complicated, since no similarities were reported to any known repeat.

The CenH3 function is similar in all species studied to date, but its protein sequence differs even between closely related species [15, 26, 27, 76, 77]. This is also the most likely reason why the anti-bvCenH3 antibody which was raised against the sugar beet CenH3 sequence failed to detect CenH3 in *Patellifolia* species. In contrast, the anti-rice CenH3 antibody recognized successfully CenH3 proteins in genera as far remote as maize and oat [51].

Sugar beet chromosomes are characterized by high enrichment of H3K9me2 in large intercalary heterochromatic blocks on each chromosome arm [13]. These intercalary heterochromatic knobs may constitute the main portion of H3K9me2 heterochromatin in sugar beet [63]. H3K9me2 ChIP-Seq in sugar beet revealed a considerable diversity of repetitive sequences. The high proportion of the pEV satellite may indicate that the pEV satellite may contribute to the establishment and maintenance of intercalary heterochromatin as has been described for satellites [13, 42]. In accordance with the model proposed by Picaard *et al.* [78] it is hypothesized that satellite DNA may contribute to the formation of heterochromatin due to epigenetic mechanisms [13, 79]: Satellites are transcribed by POLYMERASE IV into ssRNAs, and subsequently dsRNAs are generated by RNA-DEPENDENT RNA POLYMERASE. The satellite dsRNAs are processed into 24-nt siRNAs by the activity of DICER-LIKE3. 24-nt siRNAs from satellite repeats are loaded on ARGOUNAUTE4, which then recognizes POLYMERASE V satellite transcripts complementary to the incorporated 24-nt siRNA. DOMAIN REARRANGED DNA METHYLTRANSFERASE2 associates to the ARGOUNAUTE complex and induces de novo cytosine methylation at CG, CHG (H = A, C, T) and CHH sites at satellite loci homologous to 24-nt siRNAs. Furthermore, 24-nt siRNAs might be involved in the dimethylation of histone H3 [80], subsequently leading to heterochromatization as described in animals [81]. Therefore, satellites may serve as optimal sequence platform for the establishment and maintenance of heterochromatin at a large scale due to their simple structure of tandemly repeated monomers and their expansiveness of tandem arrays [79].

Similar to the enrichment in CenH3 binding sequences, the CRM families Beetle4 and Beetle7 are also found in H3K9me2 data. Several Beetle families are located beyond the centromere (e.g. pericentromeric heterochromatin). Therefore it is likely, that Beetle4 and Beetle 7 might tend to integrate beyond the centromere due to several target sites and/or the diversification steps. Based on FISH investigations [62]⁠ a similar explanation is proposed for Beetle7, which is the centromeric retrotransposon in sugar beet. It cannot be excluded that these sequences were partially located in pericentromeric heterochromatin or in centromeric chromatin, which may also contain small proportions of H3K9me2 [13]⁠. In accordance with this assumption H3K9me2 ChIP-Seq revealed small enrichment factors of 1.17 for the centromere-specific satellite pBV_III although the enrichment factor threshold chosen in this study was at least 1.5. Studies in maize also provided evidence for H3K9me2-heterochromatin in centromeres [32, 36, 37]. In immunostaining experiments very little H3K9me2 signals have been detected in sugar beet centromeres [13]. However, this faint signal intensity could also be explained by the inability of antibody or stains to access centromeres during metaphase. One reason might be the protection of centromeres by a large complex of kinetochore factors that might have not been eliminated by protease treatment of metaphase spreads. The Cotzilla family is an evolutionary young member of the SIRE clade belonging to the superfamily of Ty1-*copia* retrotransposons, which is remarkably abundant in H3K9me2 ChIP-Seq data. Although the ranking of Cotzilla enrichment factors might suggest intermediate specificity to H3K9me2 heterochromatin, ChIP-Seq data validated that Cotzilla elements constitute high proportions of H3K9me2 heterochromatic sequences. This emphasizes the findings of Weber *etal.* [38]⁠, where FISH experiments using an LTR-specific Cotzilla1 probe demonstrated its high abundance in sugar beet intercalary and pericentromeric heterochromatin. Bongo3 is a sugar beet Ty3-*gypsy* retrotransposon of the Tekay clade of chromoviruses which was found dispersed in small clusters along chromosomes [39]. Although the enrichment shows reduced specificity, Bongo3 is another element associated with H3K9me2.

Belonging to Ty3-*gypsy* retrotransposons, Elbe2, which is a sugar beet specific Errantivirus [43], exhibited a wide distribution on all chromosomes in FISH experiments. The sugar beet Elbe group includes *gag-pol* polyproteins of the elements Elbe1, Elbe2, Elbe3 and Elbe4. No restriction to certain genome regions has been reported [43]⁠. Unlike Bongo3, Elbe2 exhibits higher enrichments in H3K9me2 ChIP-Seq experiments indicating a more specific relation between Elbe2 and H3K9me2. In addition, besides satellite repeats and LTR retrotransposon sequences, the DNA transposon superfamily En/Spm was remarkably enriched in H3K9me2 ChIP-Seq data. To date, little is known about the precise localization of the transposon superfamily CACTA-En/Spm in sugar beet [82]⁠. Nevertheless, the detection of En/Spm sequences in H3K9me2 ChIP-Seq experiments is plausible as H3K9me2 is found in association with smaller heterochromatic spots in genic and transposon-rich regions [34]⁠. Interestingly, the most specific (but not most abundant) H3K9me2 binding sequence is not homologous to known repeats (Additional file 3: Table A3). Thus, the highest enrichment was found for a repeat, which has not been annotated so far. Similar to the unknown sequences in CenH3 ChIP-Seq, the characterization has to be performed to reveal the heterochromatin specificity of unknown repeats.

**Fig. 6:**
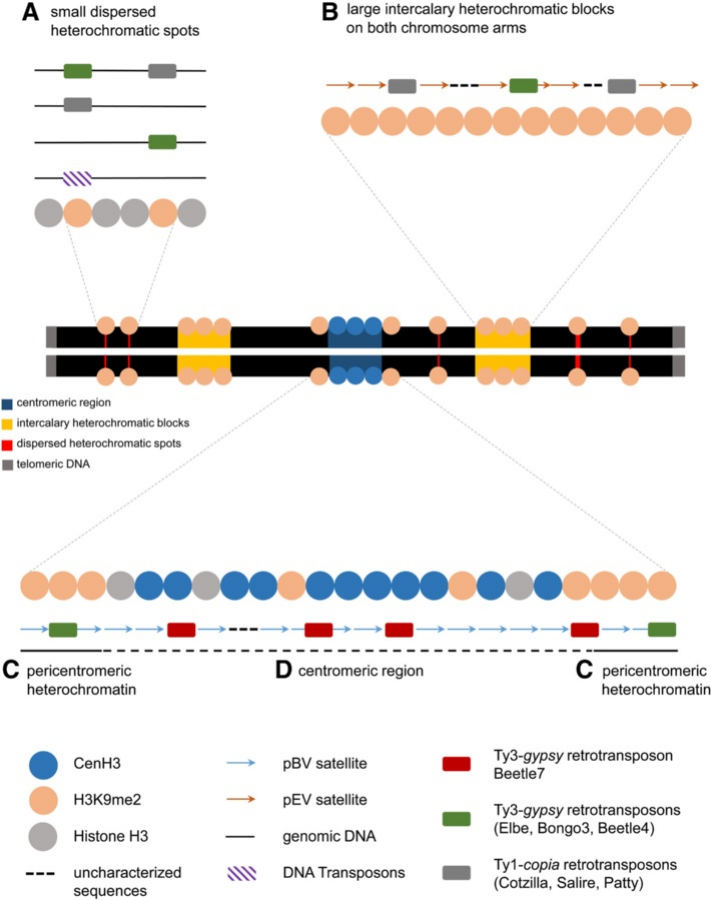
Simplified model of histone modifications/substitutions in relationship with major repeats in CenH3-chromatin and H3K9me2-heterochromatin. A typical metacentric sugar beet chromosome composed of two chromatids is illustrated. **a** H3K9me2 is found in small heterochromatic sites in genic regions, in vicinity to transposons and putatively with retrotransposons, in large clusters in intercalary heterochromatin (**b**) and in small proportions in CenH3 chromatin (**d**). The underlying sequence is composed of several repeats, but the satellite pEV and the Ty1-*copia* retrotransposon Cotzilla predominate the sequence composition. H3K9me2 interacts with Beetle4 and occasionally with Beetle7 in pericentromeric regions (**c**). Active centromeres are defined by CenH3, which preferentially binds to the centromeric satellite pBV and the Ty3-*gypsy* retrotransposon Beetle7. Beetle4 is found in centromeric and presumably also in pericentromeric heterochromatin, in association with both, H3K9me2 and CenH3. Note, that this figure is not drawn to scale. Plastid DNA is not incorporated, due to various integration sites, which need to be further confirmed and analyzed in subsequent studies

The major findings of this study were integrated in a summarizing figure (Fig. 6) based on the results obtained in this study and previously reported FISH data [13, 32–35, 38, 39, 43, 58–60, 83, 84]⁠. The simplified model shows the complexity of CenH3-chromatin and H3K9me2-heterochromatin repeat composition in sugar beet. While the satellites pEV and pBV are mutually exclusive, Ty3-*gypsy* retrotransposons are found in intercalary heterochromatin as well as in the centromere. For transposable elements of the En/Spm superfamily it might be likely that they are preferentially located in smaller dispersed heterochromatic spots beyond intercalary and (peri)centromeric heterochromatin.

Remarkably, plastid DNA apparently constitutes high proportions of both heterochromatic regions, although it has not been integrated in the genome assembly [3]. However, FISH using plastid DNA confirms dispersed chromosomal localization. Consequently, in contrast to earlier descriptions, plastid DNA shows no major preference for integration into certain chromosomal regions [85–87]. The existence of plastid DNA in sugar beet chromosomes may be due to multiple plastid DNA integration events which have taken place several times independently and therefore could be a frequent event rather than a single incident. Therefore, plastid DNA is left out of the summarizing figure as it has to be studied in far more detail which part of the chloroplast DNA and how many copies integrated into the sugar beet genome in future studies.

## Conclusions

Sugar beet highly repetitive sequences are largely amplified in heterochromatic regions and centromeric chromatin but are only fragmentary included in the genome sequence. Therefore, the repeat composition of sugar beet heterochromatin and centromeric chromatin was annotated and characterized using ChIP-Seq and subsequent sequence clustering approaches via RepeatExplorer: Centromeric chromatin is hallmarked by the sugar beet CenH3, which binds to pBV satellites and to elements of the Ty3-*gypsy* retrotransposon family Beetle7. This finding underpins the typical sequence composition of a typical plant centromere. Heterochromatin in plants is defined by the presence of H3K9me2. It occurs either in clusters (intercalary heterochromatin or pericentromere) or in smaller heterochromatic spots and is associated with a diverse pattern of repetitive sequences consisting of different families of satellite DNAs, Ty3-*gypsy* and Ty1-*copia* retrotransposons as well as DNA transposons.

The knowledge of repetitive sequences constituting the only poorly characterized heterochromatin and centromeric chromatin gained within this project provides so far missing data for the understanding of the organization and evolution of the sugar beet genome. Furthermore, it enhances the annotation quality of repetitive sequences, which is potentially useful for future genomic studies. The presented approach in sugar beet has already been performed in other plants before and is eligible to be applied to further plant genomes for the annotation and characterization of repeats localized in CenH3-chromatin and in heterochromatic blocks, which are not or only fragmentarily included in reference genome sequences. Consequently, the analysis of the complete repeat composition of active plant centromeres and heterochromatin will provide comprehensive knowledge on a neglected but major sequence component of plant genomes.

## Methods

### Propagation of plant material

For immunostaining experiments seeds of *B. vulgaris subps. vulgaris* (KWS Saat SE Einbeck, KWS-03062), *B. vulgaris subsp. maritima* (IPK Gatersleben accession no. 45503, origin: Italy), *B. patula* (IPK Gatersleben accession no. 35290, origin: Portugal) and *P. patellaris* (IPK Gatersleben accession no. 57667, origin: Spain) were germinated on wet filter paper in a germination box at room temperature. After three to seven days root meristems (approx. 1 cm root tips) were used for preparation of mitotic chromosomes.

For ChIP-Seq experiments *Beta vulgaris subsp. vulgaris* (KWS Saat SE Einbeck, KWS-03062) was grown in soil. Young leaves (1–3 cm) from approx. one month old plants were harvested, frozen in liquid nitrogen and stored at – 80 °C until needed.

### Immunostaining and Fluorescence-*in situ*-hybridization (FISH)

Mitotic chromatin was prepared on ice. Chromatin fixation was achieved by incubation in 4 % paraformaldehyde for 30 min under vacuum conditions followed by 10 min non-vacuum incubation. The tissue was macerated using an enzyme solution consisting of 0.7 % cellulase Onozuka R-10 (Serva), 0.7 % cellulysin cellulase (Calbiochem), 0.7 % cytohelicase (Sigma), 1 % hemicellulase (Sigma), 4 % pectinase (Sigma), 20 % pectolyase (Sigma) in enzyme buffer (4 mM citric acid, 6 mM sodium citrate in ddH_2_O, pH 4.5) for 60 – 90 min at 37 °C. A single root tip each was placed on polysine slide (Thermo Fisher) and chromosomes spread with the aid of a coverslip. Gentle tapping on the area around the sample separated the nuclei. Applying high pressure improved the separation of chromosomes. The coverslip was removed using a blade after freezing the slide in liquid nitrogen. Unspecific binding sites were blocked with 5 % BSA/0.001 % Tween 20 in PBS for one hour. Rabbit anti-bvCenH3 (from KWS Saat SE, produced based on the CenH3 peptide sequence NH2-RVKHTAARKSTTNGPRSKAQKC-CONH2, with subsequent affinity-purification of the monospecific IgG-fraction by Pineda Antikörper-Service, Berlin) diluted 1:200 in 3 % BSA/0.001 Tween 20 in PBS) incubated overnight at 4 °C in a humidity chamber. Secondary antibody, fluorescein (FITC) anti-rabbit (Roche), was applied for one hour at 37 °C (diluted 1:200 in 3 % BSA/0.001 % Tween 20 in PBS) after a washing series of 3 × 5 min in PBS. After 3 × for 5 min washing in PBS nuclei were counterstained with DAPI (4’, 6’-diamino-2-phenylindole) in antifade solution (Citifluor, 1:50 dilution) and a cover slip was mounted.

When DNA sequences were hybridized after immunostaining, no coverslip was mounted but chromosomes were repeatedly fixed and cross-linked in 4 % paraformaldehyde and 3.5 % sucrose-solution for 10 min at room temperature. Dehydration (70 % and 100 % ethanol for 3 min each) followed after washing slides in PBS for 3 × 5 min. The air-dried slides were now used for hybridization.

For FISH young leaves of sugar beet were used for the preparation of mitotic chromosomes. Before fixation in methanol:acetic acid (3:1), leaves were incubated for 2,5 h in 2 mmol/l 8- hydroxyquinoline. Fixed plant material was macerated in an enzyme mixture consisting of 2 % (w/v) cytohelicase (Sigma), 2 % (w/v) cellulase from *Aspergillus niger* (Sigma-Aldrich), 4 % (w/v) cellulase Onozuka-R10 (SERVA), 20 % (v/v) pectinase from *A. niger* (Sigma- Aldrich) and 0.5 % pectolyase from *A. japonicus* (Sigma- Aldrich) followed by dropping the nuclei suspension onto slides as described previously [88]. Probes of pBV satellite were labelled with biotin-11-dUTP (Thermo Fisher) by PCR (initial denaturation at 94 °C for 5 min, 35 cycles of 30 s denaturation at 94 °C, 30 s annealing at 55 °C, and 45 s of elongation at 82 °C followed by 5 min of final elongation at 72 °C). Primers used were EPI M13 (forward CGCCAGGGTTTTCCCAGTCACGAC, reverse AGCGGATAACAATTTCACACAGGA (Eurofins)). ChIP DNA probes of CenH3 and H3K9me2 were labelled with Digoxigenin using Dig High Prime DNA Labeling and Detection Kit (Roche) according to the manufacturer’s instructions. The plastid DNA probe (BAC 14G1, available upon request) was labelled with biotion-11-dUTP (Thermo Fisher) using Nick translation.

*In situ* hybridization was performed according to Schmidt *et al.* [9] with some modifications. On slides with preceding immunostainings no RNase treatment was performed. Chromosomes and probe were denatured in a Hybaid Touchdown-*in situ*-system (Thermo Scientific Hybaid). For detection of biotin-labelled probes and digoxigenin-labelled probes, slides were incubated with 5 ng/μl conjugated streptavidine-cyanine 3 (Cy3) and 1:75 diluted Anti-digoxigenin-fluorescein antibody (Roche) in blocking reagent (Roche), respectively, in a humid chamber for one hour at 37 °C. Chromosomes were counterstained with DAPI and mounted in antifade solution before examination with an “Axioplan 2 Imaging” microscope (Carl Zeiss) in combination with the digital FISH-analysis system “Applied Spectral Imaging”. Images were captured monochrome for each channel with the 12-bit digital camera (1280 × 1024 px), automatically aligned with the program Case data manager 5.5 (Mountain View, C.A.) and processed with Adobe Photoshop (CS5).

### Chromatin immunoprecipitation followed by sequencing (ChIP-Seq)

Sugar beet nuclei of 20 g leaf material for CenH3 ChIP and 40 g leaf material for H3K9me2 ChIP (Abcam, Cambridge, GB, catalog ab1220) were prepared according to Neumann *et al.* [54]*.* ChIP was performed with the aid of Dynabeads® Protein G Immunoprecipitation Kit (Life Technologies) with a preceding precleaning step and prolonged incubation times as described before [54]. ChIP-DNA and input DNA controls were sequenced on the Illumina HiSeq-1500 platform using multiplexing of the samples. Indexed libraries were generated using the TruSeq ChIP Sample Prep Kit (Illumina) according to the manufacturer’s instructions. Sequencing resulted in single-end reads with 138 nt in length.

### Read clustering and ChIP-Seq data analysis

Shotgun genomic paired-end Illumina reads (885,575) from *Beta vulgaris* were retrieved from the Sequence Read Archive (the study accession number ERA SRP029748). Repeat identification by similarity-based clustering of quality-filtered reads was performed using the RepeatExplorer pipeline with default parameters [56, 57]. The pipeline employs graph representation of read similarities to identify clusters of frequently overlapping reads representing various repetitive elements or their parts. In addition, it provides information about repeat quantities (estimated from numbers of reads in the clusters), information about cluster connections via paired-end reads used to identify repeats split between multiple clusters, and outputs from BLASTn and BLASTx [89]. Similarity searches to custom databases of repetitive elements and repeat-encoded conserved protein domains that aid in repeat annotation (the databases are composed of selected repetitive elements representing all major lineages of mobile elements from various plant taxa). This information was combined with similarity searches to a nucleotide database containing all annotated repeats of the genus *Beta* known to date [3, 11, 13, 39, 41–44, 63, 90, 91] and was used for final manual annotation and quantification of repeats from all clusters making up at least 0.01 % of sugar beet genome.

ChIP-Seq resulted in 280 million 138 nt long reads for ChIP-DNA and its input control samples. ChIP-Seq reads and reads from the input control samples (DNA fragments extracted from the chromatin prior to ChIP) were quality-filtered, trimmed to 50 nt and randomly sampled to get 10 million read fragments from each sample. These fragments were mapped to contigs representing assembled cluster reads using BLASTn with the parameters -m 8 -b 1 -e 1e-12 -W 9 -r 2 -q −3 -G 5 -E 2 -F F. Each read was mapped to a maximum of one cluster, based on its best similarity. Proportion of ChIP and input reads mapped to individual clusters was evaluated to identify repeats with ChIP/input ratio > = 1.5 that were considered to represent repeats enriched in the ChIP sample.

## Additional files

Additional file 1: Table A1. Genomic reference clusters of *Beta vulgaris*. (XLS 28 kb) Additional file 2: Table A2. Enriched clusters in CenH3 ChIP-Seq data. (XLS 12 kb) Additional file 3: Table A3. Enriched clusters in H3K9me2 ChIP-Seq data. (XLS 19 kb)
